# CCL21 and prognosis in acute coronary syndrome

**DOI:** 10.18632/aging.102443

**Published:** 2019-11-06

**Authors:** Thor Ueland, Pål Aukrust, Kenneth Caidahl

**Affiliations:** 1 Research Institute of Internal Medicine, Oslo University Hospital Rikshospitalet, Oslo, Norway; 2 Section of Clinical Immunology and Infectious Diseases, Oslo University Hospital Rikshospitalet, Oslo, Norway; 3 Faculty of Medicine, University of Oslo, Oslo, Norway; 4 K.G. Jebsen-Thrombosis Research and Expertise Center (TREC), University of Tromsø, Tromsø, Norway; 5 Department of Molecular and Clinical Medicine, Sahlgrenska Academy, University of Gothenburg, Gothenburg, Sweden; 6 Department of Clinical Physiology, Sahlgrenska University Hospital, Gothenburg, Sweden; 7 Departments of Molecular Medicine and Surgery and Clinical Physiology, Karolinska Institutet and Karolinska University Hospital, Stockholm, Sweden

**Keywords:** CCL21, acute coronary syndrome, prognosis, non-ST-segment elevation myocardial infarction (or NSTEMI)

The management of patients with acute coronary syndromes (ACS) has improved markedly in the last decades through the development of new biomarkers such as the troponins, natriuretic peptides and risk stratification scoring systems. However, there has been a shift in the epidemiological characteristics of myocardial infarction (MI) with an increasing number of patients presenting with non-ST-segment elevation MI (NSTEMI) where the ability to predict short- and long-term outcomes is more limited [1,2]. Exploration of novel biomarkers reflecting activated pathways not accounted for by current risk markers could improve diagnosis and prognostic classification of patients with ACS and identify new targets for therapy.

Chemokines are small heparin-binding proteins that together with their chemokine receptors are critical mediators of cell migration during immune surveillance and homeostasis. The homeostatic chemokines, CCL19 and CCL21 and their common receptor CCR7, play a pivotal role in T cell and dendritic-cell trafficking into lymphoid tissue, and their function was originally thought to be restricted to immune cell homeostasis. However, findings during the last decade suggest that these chemokines may have pleiotropic effects and are involved in inflammatory responses in non-lymphoid tissue such as atherosclerotic lesions implying a complex role for CCR7 signaling in immune responses [3]. We and others have shown that CCL19/CCL21 may contribute to atherogenesis by different mechanisms including enhanced recruitment of T cells and macrophages to atherosclerotic lesions and induction of a matrix degrading, pro-thrombotic and inflammatory phenotype in these and other vascular cells [3,4]. CCR7 activation seems also to contribute to foam cell formation in macrophages. Thus, elevated circulating levels of CCL19 and CCL21 have been demonstrated in patients with atherosclerotic heart disease [3,4]. Furthermore, experimental MI models are characterized by higher cardiac expression and circulating levels of CCL21 [5], and in a clinical setting CCL21 was associated with adverse outcomes in acute and chronic heart failure, and in aortic stenosis [5,6]. Thus, in the setting of ACS, CCR7/CCL19/CCL21 signaling may promote plaque development and rupture, but could also play a role in cardiac remodeling following MI.

**Figure 1:**
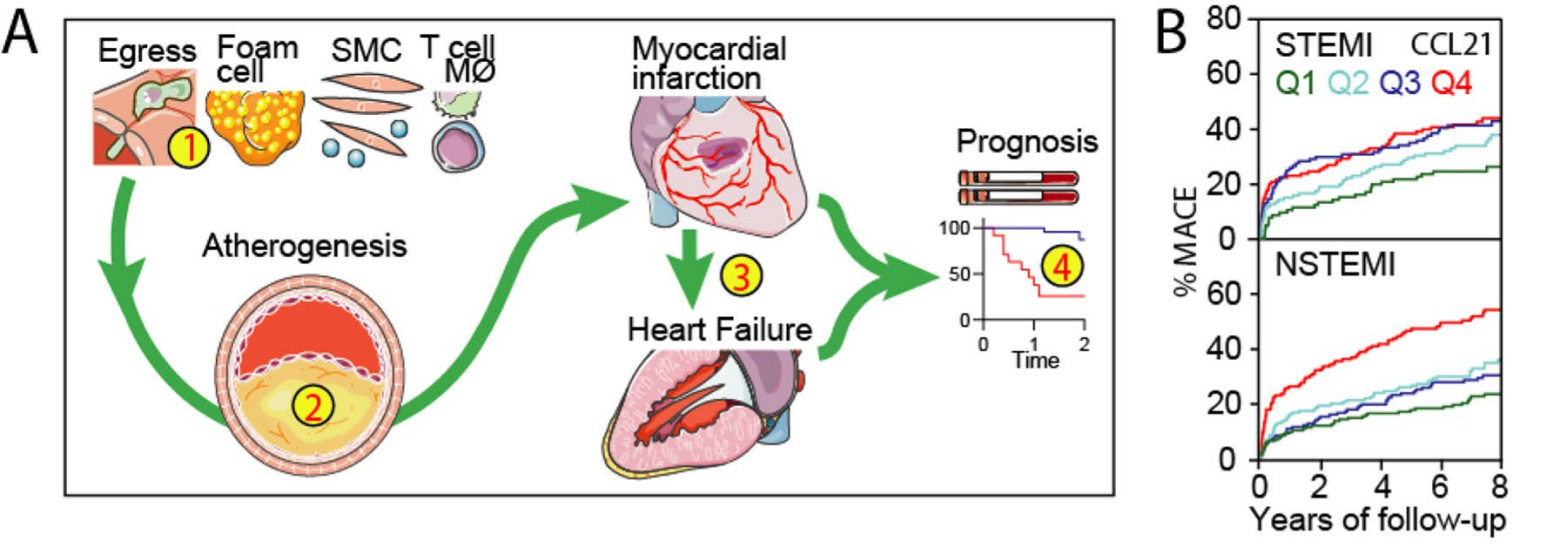
**CCL21 and prognosis in acute coronary syndromes.** (**A)** (1) CCL21 may contribute to atherogenesis by different mechanisms including recruitment of T cells and macrophages to atherosclerotic lesions and induction of a matrix degrading, pro-thrombotic and inflammatory phenotype in these and other vascular cells leading to atheroma formation (2) and potentially thrombosis and myocardial infarction (3). CCL21/CCr7 interactions are also involved in adverse cardia remodeling and high circulating levels (4) are associated with poor outcome in patients with cardiac dysfunction. (**B)** We recently reported that CCL21 was associated with MACE following ACS and further analysis shows that this association is most prominent in NSTEMI.

Recently, we demonstrated in a large population of ACS patients that high serum CCL21 was associated with short- and long-term major cardiovascular events (MACE) after full multivariable adjustment including troponins, natriuretic peptides and GRACE risk score [7]. The role of CCL21 in atherogenesis and prognosis in cardiovascular disease is shown in Figure 1A. CCL21 levels were not correlated with troponin levels but were associated with Killip class and GRACE risk scores and the association with long-term MACE was primarily driven by a high risk of MI suggesting that CCL21 reflects plaque progression and instability rather than the degree of cardiac injury. As shown in Figure 1B, further analysis of this cohort (unpublished data; Ueland, Aukrust and Caidahl) shows that this association was most pronounced in NSTEMI patients with an adjusted HR and 95% CI for quartile 4 of CCL21 reaching 1.70 (1.16-2.50) p=0.006 compared to HR 1.08 (0.71-1.66) p=0.71 in STEMI.

The shift in presentation of ACS with more frequent NTSEMI coincides with a change in the character of atherosclerotic plaques with less lipids and inflammation and more fibrous tissue and interaction between neutrophils and endothelial cells that are vulnerable to superficial erosion rather than plaque rupture [1]. Matrix degradation and platelet activation seem also to play a central role in these processes. While current treatment of ACS has been effective in counteracting plaque ruptures, novel treatment modalities may be needed to reduce plaque erosion that seems to play a major role in the pathogenesis of NSTEMI. The higher incidence of MACE in NSTEMI patients with high CCL21 levels in our study could indicate a role for CCR7/CCL21 interaction in plaque erosion. Indeed, CCR7 plays an important role in migration, activation and survival of multiple cell types including neutrophils and endothelial cells, and we have also shown that CCR7 could modulate vascular smooth muscle cells to a proliferating and matrix degrading phenotype which clearly is relevant for plaque erosion [8].

Finally, CCL21 was strongly correlated with cardiac dysfunction and natriuretic peptide levels supporting a role for CCL21 in maladaptive remodeling [5]. Both knockdown of CCR7 and anti-CCL21 treatment suppressed post-MI remodeling and improved survival and cardiac function in experimental MI suggesting that anti-CCL21 strategies could be a potential treatment options also following ACS.

We suggest that the CCR7/CCl21/CCL19 interaction could be an interesting molecular axis in ACS, not only as a novel and promising biomarker in these patients and in particular in NSTEMI patients, but also as a novel therapeutic target attenuating not only plaque destabilization, but also development and progression of plaque erosion and maladaptive remodeling following MI.
